# Physician Awareness of Drug Cost: A Systematic Review

**DOI:** 10.1371/journal.pmed.0040283

**Published:** 2007-09-25

**Authors:** G. Michael Allan, Joel Lexchin, Natasha Wiebe

**Affiliations:** 1 Department of Family Medicine, University of Alberta, Edmonton, Alberta, Canada; 2 Institute of Health Economics, Edmonton, Alberta, Canada; 3 Department of Family and Community Medicine, University of Toronto, Toronto, Ontario, Canada; 4 School of Health Policy and Management, York University, Toronto, Ontario, Canada; 5 Department of Medicine, University of Alberta, Edmonton, Alberta, Canada; World Health Organization, Switzerland

## Abstract

**Background:**

Pharmaceutical costs are the fastest-growing health-care expense in most developed countries. Higher drug costs have been shown to negatively impact patient outcomes. Studies suggest that doctors have a poor understanding of pharmaceutical costs, but the data are variable and there is no consistent pattern in awareness. We designed this systematic review to investigate doctors' knowledge of the relative and absolute costs of medications and to determine the factors that influence awareness.

**Methods and Findings:**

Our search strategy included The Cochrane Library, EconoLit, EMBASE, and MEDLINE as well as reference lists and contact with authors who had published two or more articles on the topic or who had published within 10 y of the commencement of our review. Studies were included if: either doctors, trainees (interns or residents), or medical students were surveyed; there were more than ten survey respondents; cost of pharmaceuticals was estimated; results were expressed quantitatively; there was a clear description of how authors defined “accurate estimates”; and there was a description of how the true cost was determined. Two authors reviewed each article for eligibility and extracted data independently. Cost accuracy outcomes were summarized, but data were not combined in meta-analysis because of extensive heterogeneity. Qualitative data related to physicians and drug costs were also extracted. The final analysis included 24 articles. Cost accuracy was low; 31% of estimates were within 20% or 25% of the true cost, and fewer than 50% were accurate by any definition of cost accuracy. Methodological weaknesses were common, and studies of low methodological quality showed better cost awareness. The most important factor influencing the pattern and accuracy of estimation was the true cost of therapy. High-cost drugs were estimated more accurately than inexpensive ones (74% versus 31%, Chi-square *p* < 0.001). Doctors consistently overestimated the cost of inexpensive products and underestimated the cost of expensive ones (binomial test, 89/101, *p* < 0.001). When asked, doctors indicated that they want cost information and feel it would improve their prescribing but that it is not accessible.

**Conclusions:**

Doctors' ignorance of costs, combined with their tendency to underestimate the price of expensive drugs and overestimate the price of inexpensive ones, demonstrate a lack of appreciation of the large difference in cost between inexpensive and expensive drugs. This discrepancy in turn could have profound implications for overall drug expenditures. Much more focus is required in the education of physicians about costs and the access to cost information. Future research should focus on the accessibility and reliability of medical cost information and whether the provision of this information is used by doctors and makes a difference to physician prescribing. Additionally, future work should strive for higher methodological standards to avoid the biases we found in the current literature, including attention to the method of assessing accuracy that allows larger absolute estimation ranges for expensive drugs.

## Abstract 2

From a review of data from 24 studies, Michael Allan and colleagues conclude that doctors often underestimate the price of expensive drugs and overestimate the price of those that are inexpensive.

## Editors' Summary

**Background.:**

Many medicines are extremely expensive, and the cost of buying them is a major (and increasing) proportion of the total cost of health care. Governments and health-care organizations try to find ways of keeping down costs without reducing the effectiveness of the health care they provide, but their efforts to control what is spent on medicines have not been very successful. There are often two or more equally effective drugs available for treating the same condition, and it would obviously help keep costs down if, when a doctor prescribes a medicine, he or she chose the cheapest of the effective drugs available. This choice could result in savings for whoever is paying for the drugs, be it the government, the patient, or a medical insurance organization.

**Why Was This Study Done?:**

Doctors who prescribe drugs cannot be expected to know the exact cost of each drug on the market, but it would he helpful if they had some impression of the cost of a treatment and how the various alternatives compare in price. However, systems deciding how drugs are priced are often very complex. (This is particularly the case in the US.) The researchers wanted to find out how aware doctors are regarding drug costs and the difference between the alternatives. They also wanted to know what factors affected their awareness.

**What Did the Researchers Do and Find?:**

They decided to do a systematic review of all the research already conducted that addressed this issue so that the evidence from all of them could be considered together. In order to do such a review they had to specify precise requirements for the type of study that they would include and then comprehensively search the medical literature for such studies. They found 24 studies that met their requirements. From these studies, they concluded that doctors were usually not accurate when asked to estimate the cost of drugs; doctors came up with estimates that were within 25% of the true cost less than one-third of the time. In particular doctors tended to underestimate the cost of expensive drugs and overestimate the cost of the cheaper alternatives. A further analysis of the studies showed that many doctors said they would appreciate more accurate information on costs to help them choose which drugs to prescribe but that such information was not readily available.

**What Do These Findings Mean?:**

The researchers concluded that their systematic review demonstrates a lack of appreciation by prescribing doctors of the large difference in cost between inexpensive and expensive drugs, and that this finding has serious implications for overall spending on drugs. They call for more education and information to be provided to doctors on the cost of medicines together with better processes to help doctors in making such decisions.

**Additional Information.:**

Please access these Web sites via the online version of this summary at http://dx.doi.org/10.1371/journal.pmed.0040283.

- A brief guide to systematic reviews has been published by the *BMJ (British Medical Journal)*
- The Web site of the Cochrane Collaboration is a more detailed source of information on systematic reviews; in particular there is a newcomers' guide and information for health-care consumers
- The Kaiser Family Foundation, a nonprofit, private operating foundation focusing on the major health care issues in the US, has a section on prescription drugs and their costs

## Introduction

Financial constraints are a reality in almost all aspects of medicine. Pharmaceutical expenditure ranges from 8.5% to 29.6% of health-care spending within Organisation for Economic Co-operation and Development countries and is increasing faster than other areas of health-care spending in almost all these countries [1]. For example, in Canada pharmaceutical spending increased from 9.5% of total health care costs in 1985 to over 16% in 2004, and its annual growth rate has exceeded that of all health expenditures in every year in that period [2]. Most countries struggle to reduce pharmaceutical spending [3,4] as escalating costs and limited resources threaten other budgetary priorities. While the policy makers in publicly funded systems and insurance agencies struggle to cope, strategies to shift costs, in part or whole, to the consumer are unavoidable. Unfortunately, these initiatives often shift costs to other areas of health care, result in worse patient outcomes, and are not cost-effective overall [5–10]. Initiatives that have targeted doctors to reduce pharmaceutical spending include guidelines, fund-holding, and others [11–13]. One way of helping to control drug costs would be for physicians to autonomously choose the least-costly medication when there are no substantial differences in safety and effectiveness between the least and most expensive. Price variations within drug classes [14,15] or between drug classes are common, and if physicians were to choose therapeutically equivalent but less-expensive drugs, large scale savings could be realized.

In addition to budget concerns, doctors must consider drug costs to their patients. Increasing pharmaceutical costs negatively impacts patients in two ways. First, high direct expenses for those of limited resources may mean a choice between medicines and necessities such as food or clothing [16,17]. Alternatively, patients who do not take their medicine as directed or go without the potentially beneficial therapies entirely [16,17] often suffer negative health consequences [5–8,10]. Unfortunately, patients may be too embarrassed to tell their physicians when they cannot afford their medicines [18,19].

### Background: Drug Costs and Patient Expenses

In the global market, the cost of drugs is highly variable and therefore obtaining accurate and relevant costs is often very complex. The situation in the United States (US) is likely the most complex, and multiple authors have attempted to distil the confusing and convoluted story of drug costs [20,21]. The often-quoted average wholesale price (the distributors' price to pharmacies) can vary due to multiple factors such as demand, recent negotiations with pharmaceutical manufacturers, and changes in coverage from large insurers. At the pharmacy, mark-up of the average wholesale price can be dramatic depending on the type of product (acute medicines have a larger mark-up) or the method of payment (cash customers often pay more). Alternatively, some high-use drugs may be marked down to draw customers in to the store. The amount the patient pays is based on his or her insurance, through private organizations such as managed care organizations and health maintenance organizations, government support (for example, Medicaid), or a combination of the above (families may have two or more providers). Insurers use a wide variety of strategies to control costs including copayment, tiered copay (the amount of shared payment varies with different drugs), and reference pricing for drugs, to name a few. Each insurer (private or government) covers different drugs and have different copay systems (flat fees, percent copay, or a mixture of the two).

Elsewhere, the system is slightly less complex. In Canada, drug prices are believed to more closely parallel the wholesale price but are still subject to some of the variations and price competition found in the US. There is provincially based drug coverage for seniors and low-income individuals, but many provinces have some form of copay or reference -based pricing. The rest of the population pays for drugs out of pocket or has some form of insurance (which frequently has a copay component). In Europe, there are dramatic (>400%) differences in drug costs between neighboring countries [22,23]. Many countries have some elements of price competition (e.g., United Kingdom [UK] and Germany), but in some countries companies negotiated costs with regional (e.g., Spain) or federal (e.g., Italy) governments [24]. Many countries, including UK, France, Germany, Italy, The Netherlands, Spain, Finland, Denmark, and Austria, have some form of copay [3,23]. The copay systems are often added to a mix of complementary insurance (e.g., France), reference-based pricing (e.g., The Netherlands), drug budgets for physicians (e.g., UK), price control (e.g., Italy) and combinations of them all with regional variation in some countries [3]. The systems are at times irrational. For example, fixed copayments in some countries can result in patients paying more for a prescription than the actual list price of the drug [23]. Although many North Americans believe that drugs are free to patients in Europe, copayments have been shown to be a barrier for patients even in the UK [19]. Many other countries (e.g., Australia and Japan) also use a variety of copayment or cost-sharing schemes for prescriptions [24,25].

Therefore, with global budgets a concern and the welfare of patients at risk, physicians need to consider drug cost when prescribing. If physicians are going to take costs into consideration they need to be cognizant of both the absolute drug cost and the relative differences between prices of products. However, in most places cost information is not easily available for doctors and even where it is, the large difference between inexpensive and expensive equivalents is not emphasized. To determine if it is necessary to enhance both physicians' education about prices and the availability of that information, we undertook a systematic review to determine physicians' level of awareness of the cost of prescription drug products.

## Methods

Templates for systematic review of survey studies are not well established, but QUOROM [26] (normally reserved for systematic reviews of randomized controlled trials) is a good guide for most systematic reviews and was used here wherever possible (Table S1).

### Search

We searched the Cochrane Library (from 1966), EconLit (from 1969), EMBASE (from 1974), and MEDLINE (from 1950) up to 31 May 2005 using the search terms “physician”, “doctor”, “medical student”, “house staff”, “intern” or “resident”; “medicine”, “medications”, “drug”, “therapeutic”, “test”, “investigation” or “diagnostic test”; “cost” or “price”; and “knowledge”, “awareness” or “understanding”. The original search attempted to capture all cost awareness studies including those in which doctors estimated the costs of investigations (knowledge of the cost of investigations will be presented in another publication). The titles and abstracts, where available, were independently screened by GMA and JL and if either investigator thought that the article would be potentially eligible, a complete copy was obtained. To identify additional studies, the reference list of any potentially eligible article was searched and authors with two or more publications in the area or who had published in the 10 y preceding the start of our review were contacted.

### Eligibility

Articles were included if: either doctors, trainees (interns or residents), or medical students were surveyed; there were more than ten survey respondents; costs of pharmaceuticals were estimated; results were expressed quantitatively; there was a clear description of how authors defined “accurate estimates”; and there was a clear description of how the true cost was determined. Because costs are variable and complex, we felt it was only reasonable for doctors to have knowledge of the total costs of the prescription, whether that cost was borne partially or completely by the patient and/or the insurer (private or government), in their local practice environment. Therefore, “true cost” was operationally defined as the actual cost the study authors verified from one or more locally relevant reliable sources for each drug in their study. This source would vary by location, but in the UK drug prices are more uniform so the British National Formulary would be a reasonable source, while in US quotes from local pharmacies (averaged from a broad sample) is most appropriate [21]. The definition of “accurate estimates” was taken from the authors and typically fell within a defined “accuracy range” (e.g., ±25%) around the true cost. Articles were excluded if they were not published in English or if participants were asked to estimate costs within ranges or cost increments only (for example “please estimate which $20 cost category/range is most appropriate for drug A”). GMA and JL independently assessed each potential article for eligibility. Differences in decisions about inclusion and exclusion were resolved through consensus.

### Data Extraction

From each eligible article GMA and JL independently extracted the following information: publication year; study country; response rate and number of participants, sample selection method (random, entire specified population, convenience); mode of survey administration (postal, hospital mail, meeting, face-to-face); participant level of training (medical student, intern, resident, qualified doctor); specialty; number of different drugs estimated; method of ascertainment of true cost (from formulary, acquisition cost, amount billed to patient, survey of retail pharmacies, wholesale price); method of assessing accuracy of cost estimate (within a specified percent or dollar range of true cost); and estimation accuracy (percent of respondents with accurate estimate, percent above and below true cost, median percent error of estimations). Primary quality measures were method of sample selection, mode of survey administration, and response rate, as well as errors or unclear description of calculations (e.g., incorrect method of calculating estimation error). This selection was based on our understanding of the places where the greatest biases can occur in survey studies. Where data were not reported in a way that allowed extraction in one of our categories, we attempted to calculate the information from available data (e.g., number of respondents calculated from the number of surveys distributed multiplied by the response rate). Comparisons within studies, such as differences between medical student and resident accuracy, were extracted when available. Qualitative information, such as surveys of physicians' opinions, was also extracted when available. Authors were contacted for further data where necessary. After each investigator independently extracted the above information, the results were compared and differences resolved by consensus.

### Data Analysis

The studies were too diverse to pool meta-analytically (e.g., different therapies, different cost estimation procedures, different groups of physicians), but we did examine accuracies by grouping studies with nonparametric summaries. Mean accuracy (expressed as the percent of physicians who correctly estimated drug costs) for each study was calculated by averaging the accuracy from each participant group or drug estimated with weighting for the number of estimation attempts. For example, if accuracy was 30% for drug A (*n* = 100) and 50% for drug B (*n* = 80), the average accuracy would be 39% ([(0.30 × 100) + (0.5 × 80)] ÷ 180). We calculated nonparametric summaries (median and ranges [minimum – maximum]) for the following outcomes: average cost accuracy (within defined percent margins of error), average percent of estimates over and under true cost, average percent of estimates over and under the margins of error (as defined by the original authors) around the true costs, and average percent error (|estimate – true cost|/true cost).

Percent error is the statistic used to demonstrate the degree of estimation error. To be reliable, each estimate error (the amount above or below the true cost) must be converted to an absolute value. If it is not, high estimates will be positive numbers and low estimates will be negative numbers, and when summed will partially cancel each other giving a lower value and a false impression of accuracy. For example, if the true cost of a drug is $100 a month and two doctors estimate $50 and $150 respectively, the correct percent error would be 50%. However, if absolute values were not used, the percent error of the high estimation error would be 50% and the low would be −50%. This would make the combined percent error 0%, indicating no error in estimation and yielding a false representation of perfect accuracy.

Additionally, a priori-defined subgroups, such as year of publication (divided by median year of publication of studies), location of study, training level of participants, and specialty were examined to determine if these variables influenced the accuracy of the cost estimation. We also examined the influence of study quality on estimation accuracy by separating studies with a similar accuracy range into those of high, mid, and low quality. For this analysis, we used weaknesses of response rate (≤50% or unclear), sampling method (convenience or unclear), and survey distribution (unclear) as markers of quality. While there is no defined adequate response rate, low response rates can bias surveys [27,28] and we felt 50% was generous. Nonprobability sampling, such as convenience sampling, can bias studies because the sample is not representative of the population. Different modes of questionnaire administration have different inherent biases, and while there is no clearly superior method [29], we felt the information was important in reviewing surveys. High-quality studies had none of these weaknesses, mid-quality studies had a single weakness, and low-quality had two or more weaknesses. In post hoc analyses, where studies reported potential within-study factors influencing the accuracy of cost estimation (e.g., cost of drug), we used the binomial test to combine “votes” across studies.

We also performed two sensitivity analyses. To minimize the heterogeneity inherent to comparing studies with multiple different drugs, we compared the average cost accuracies for specific drugs common among three or more studies. When data cannot be combined and nonparametric statistics such as medians and ranges must be used, there is a concern that larger studies are weighted equally with smaller ones. To determine the potential influence of “weighting,” we performed sensitivity analyses where the median nonparametric statistic was selected based on the number of therapies in each study, the number of physicians in each study, or the total number of estimates in each study.

Ethics approval was not required as the research involved publicly available material.

## Results

### Literature Search and Study Selection

**Figure 1:**
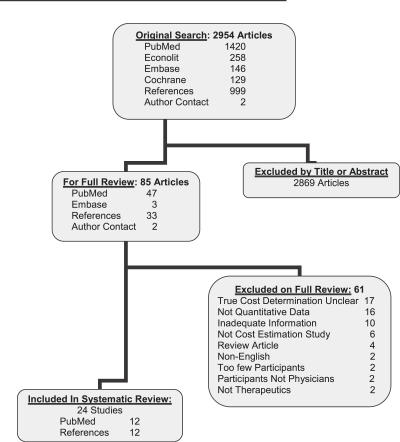
Study Identification and Selection Process

A study flow diagram is provided in Figure 1. Eleven authors were contact to identify possible studies and six responded, to yield two previously unidentified studies. From a total of 2,954 studies, 24 were included in the systematic review (Table S2 provides the list of articles excluded after full review and the reason for exclusion). Disagreement between reviewers was rare (2% in eligibility and 6% in data extraction).

### Study Characteristics

The main characteristics and methodological aspects of each study are provided in Table 1. Studies were conducted from 1978 to 2004 in six countries, with the US (nine studies), UK (eight), and Canada (four) predominating. Eleven studies included licensed physicians only, two involved house staff only, and 11 included a mixture of participants. Eight studies involved general practitioners (GPs) alone, seven specific specialists groups, six a mix, and three were unclear as to the specialty of the doctors.

**Table 1:**
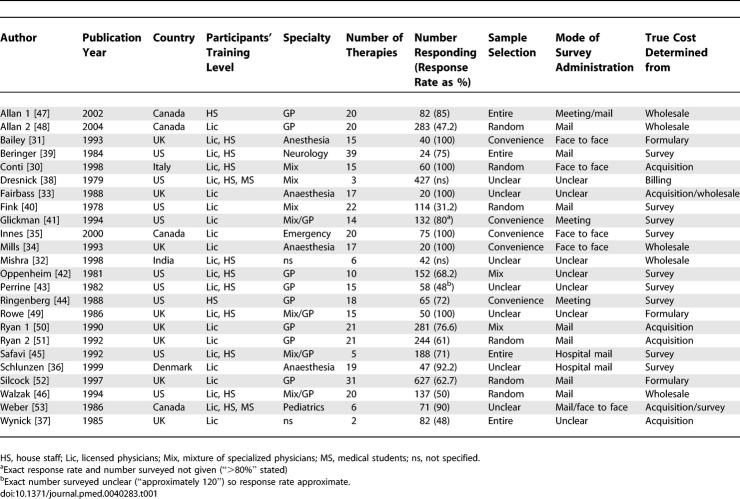
Study Characteristics

Hospital-based studies in Canada, Denmark, Italy, India, and the UK defined true costs with formulary lists from the hospital [30], government formularies [31,32], wholesale costs paid by the hospital [33,34], or total cost to the hospital [35–37]. The only US study of in-hospital physicians used hospital charges as true costs [38]. Most US outpatient studies [39–45] used the averaged prices from surveys of local pharmacies to determine true costs, but one [46] used the average wholesale price. Most of the remaining outpatient studies from Canada and the UK, where price varies little from the single-payer agreed reimbursement, determined true cost from single sources such as the wholesale costs [47,48] or the British National Formulary [49–52]. One Canadian study used a pharmacy survey for outpatient prescribing and cost to the hospital for inpatient prescribing [53].

The majority of studies (79%) selected drugs based on the common drugs for that specialty. The others picked agents based on specific representative mixes of generic/branded medications [41,50–52] or based on cost impact by frequency and expense [35]. Only two studies specifically identified the percent of generics (30% [46] and 39% [52]), but it appears the proportion in studies overall was approximately 50%.

### Study Quality

Quality and methodological reporting were poor in most of the studies. The method of survey distribution was unclear in seven studies, and sampling was convenience or unclear in 12 studies. The response rates were ≤ 50% or unknown in seven studies. Only seven (29%) of 24 studies [30,39,45,47,50–52] did not have any of these three weaknesses. In addition, of 12 studies attempting to quantify the degree of estimation error (for example percent error), nine used average estimations without regard for signage (that is, averaging overestimates with underestimates) or inadequately described the calculation. In total, 19 (79%) of the 24 studies had one or more of these four weaknesses, and only five trials [30,39,47,50,51] were without substantial weaknesses. There was also a large variation in study design; five methods were used to determine true costs, and reasonable accuracy was defined nine different ways.

### Estimation Accuracy

Table 2 summarizes cost estimation accuracy outcomes. In general, average estimation accuracy was less than 50%, decreasing with tighter definitions of accuracy. Overestimation tended to be more frequent than underestimation, and percent error was very large (well over 200%). In the sensitivity analyses of the most commonly used margin of error (±20% or ±25%), the number of therapies, the number of physicians, or the number of estimates from each study did not change the median cost accuracy by more 2%. This finding demonstrates that weighting would not have influenced the final result and that the median accuracy is very similar to a weighted mean if the data could have been combined.

**Table 2:**
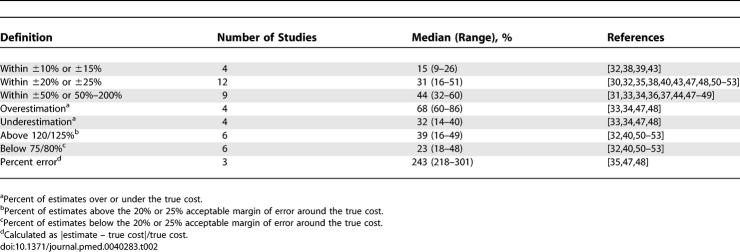
Cost Accuracy Summaries

Table 3 presents nonparametric summaries for subgroups using the most commonly used margin of error (±20% or ±25%). Results were similar using the ±50% or 50%–200% margin of error (unpublished data). While dramatic differences were not apparent, the quality of the studies may play a role in reporting the accuracy of cost estimation. By comparison, the highest-quality studies had a median accuracy of 29% (range 16%–33%) while the lowest quality studies had a median accuracy of 38% (range 27%–45%).

**Table 3:**
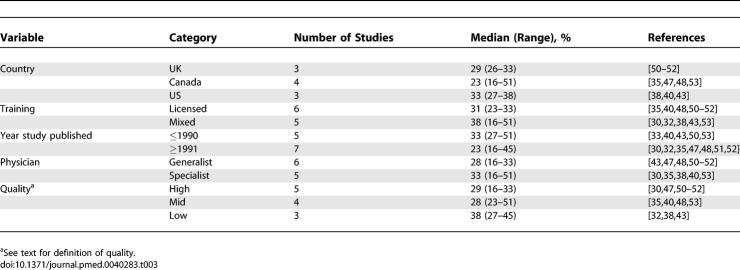
Between-Study Comparisons in Cost Accuracy (for Studies Using 20% or 25% Margins of Error for Accuracy)

**Figure 2:**
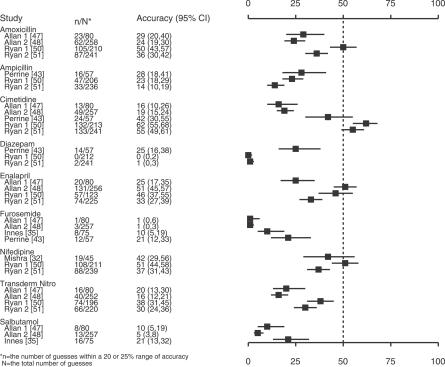
Comparison of Accuracy for Similar Drugs Comparison of estimation accuracy (within 20% or 25% margin of error) of the same drug among different studies.

There is large estimation variability within studies (percent error), and between studies accuracy varied widely (for studies using a 20% or 25% margin, average accuracy ranged from 16% to 51%). When some heterogeneity is reduced by focusing on estimation accuracy for the same drugs, the variability between studies persists (Figure 2).

### Factors Influencing Estimation Pattern and Accuracy

Table 4 summarizes the results of subgroup comparisons within included studies. Very few variables impacted the estimation of cost. Two studies [39,42] of three found cost estimations of nonacademic physicians more accurate than academic physicians. The most consistent factor influencing the pattern of estimation was the true cost of the therapy. All 11 studies that examined the influence of drug price on estimation patterns found that expensive drugs are consistently underestimated and inexpensive drugs are consistently overestimated. This finding was reinforced in the five studies [35,47,48,50,51] that provided enough data (true cost and the percentage of high/low estimations for each drug) to examine the effect of drug cost on the estimation pattern for individual drugs. For 89 of the 101 drugs in these studies, doctors consistently overestimate the cost of inexpensive drugs and underestimate the cost of expensive drugs (binomial test, 89/101, *p* < 0.0001).

**Table 4:**
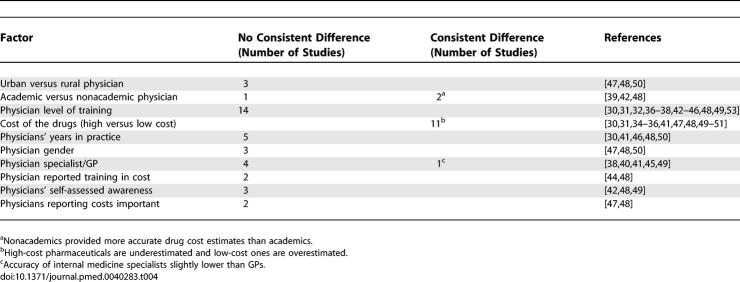
Impact of Different Factors on Accuracy of Cost Estimation

Six studies [35,43,47,48,50,51] provided enough data (true cost and estimation accuracy for each drug) to examine the implication of the true drug cost on the estimation accuracy for individual drugs. Expensive drugs are generally estimated more accurately: compared to the mean estimation accuracies for the studies, only 23 (31%) of 74 inexpensive drugs had a higher estimation accuracy while 32 (74%) of 43 of expensive drugs had a higher estimation accuracy (Chi-square, *p* < 0.001).

The influence of physician membership in a health maintenance organization or managed care organization is uncertain because US outpatient studies involved large communities and did not specifically examine or identify physicians within these organizations. One UK community study [52] compared estimation accuracy of fund holders to non-fund holders and users with desktop computer cost information to those without and found no difference except for a slightly (2%, *p* = 0.01) improved awareness among fund holders for inexpensive and very inexpensive drugs. However, the authors acknowledge that weaknesses in the computer program may have limited the utility of cost information, and the higher awareness may be due to selection bias because physicians chose to be fund holders and likely had a prior interest in costs [52].

### Qualitative Information

Many of the studies collected additional qualitative cost information. When asked, doctors rated their cost awareness as low in five of five studies [39,40,45,46,48], rated their previous cost education as low or absent in five of five studies [30,39,42,44,48], and stated that costs are important in eight of eight studies [41,42,45–48,50,52]. In four of four studies doctors reported that cost information is not easily accessible [41,46,48,50] but that they wanted more cost information [45,46] and that it would change their prescribing [48,50,52] without negatively impacting patient care [50,52], or would improve patient care [46].

## Discussion

Physicians' awareness of the cost of therapeutics is poor. With only 31% of estimates within 20% or 25% of the true drug cost and the median estimate 243% away from the true cost, many of the estimates appear to be wild guesses. Country, level of training, specialty, and other factors seem to have little impact on the degree of awareness. Despite substantial and increasing concern about costs, doctors' awareness has not improved in the 26-year span of these studies. Estimation accuracy does not appear to differ among the three countries—Canada, UK, or US—where the vast majority of the studies were conducted nor in the other countries (India, Denmark, Italy) represented in the included studies. Direct comparison of awareness between countries is limited because no study simultaneously surveyed doctors from different countries. Comparison of the estimation accuracy for the same drug across different studies showed persistent inaccuracies in the estimation patterns. This pattern suggests that removing some of the heterogeneity does not improve estimation variability, and poor awareness of cost is pervasive among physicians.

Common wisdom would have it that doctors' knowledge of drug costs is poor, and at this level our study seems to add little to what is already known. However, what is unique to our systematic review is the finding that no factors influence physicians' drug price awareness or estimation pattern except for the cost of the drug. The demonstration that estimation accuracy improves with higher-cost drugs is almost certainly not due to improved awareness of the cost of expensive drugs, but simply reflects the method used to measure awareness that grants larger absolute margins of error for more-expensive drugs. For example, when accuracy is defined as within 25% of the true cost, doctors' estimations have to be within $0.25 of a drug that cost $1.00 a month to be accurate, whereas they could be $25 off from a $100-per-month drug and still be accurate. While this finding should not be interpreted as physicians having better cost awareness of expensive drugs, an understanding of the bias in this method of assessing accuracy is helpful in explaining the variability in accuracy within and between studies. For example, from Figure 2 we can see that estimation accuracy is highly variable for cimetidine and that it is estimated more accurately in Ryan 1 [50], Ryan 2 [51], and Perrine [43] than in Allan 1 [47] and Allan 2 [48]. Cimetidine was the second most expensive drug in Ryan 1 [50] and Ryan 2 [51] and the most expensive in Perrine [43], allowing larger margins of absolute error. In Allan 1 [47] and Allan 2 [48] cimetidine was a low-cost drug and therefore had narrower margins of error. Correspondingly, studies can appear to have higher accuracies due to higher proportions of expensive drugs.

Perhaps the most valuable finding of our review is how a drug's cost influences the pattern of a doctors' estimation and how that reflects doctors' understanding of drug costs. In the movement to contain health-care costs it is extremely important to recognize the consistent lack of appreciation of the large difference in cost between inexpensive and expensive drugs. The erroneous perception of minimal differences in true costs could have profound implications for overall drug expenditures. Even if a doctor was concerned about costs and was aware that one drug was more expensive than another, he or she might still choose that expensive drug because of a belief that the cost difference is small. For example, if the doctor of a patient with uncomplicated hypertension does not realize the large price difference between the high- and low-cost products (e.g., in the province of Ontario the daily cost for hydrochlorothiazide, a thiazide diuretic, is CAN$0.01 versus CAN$1.05 for valsartan, an angiotensin 2 receptor antagonist), she or he may prescribe the more-expensive agent. In this case, there is a 100-fold difference in prices, and over ten years this difference would amount to additional expenditure of almost CAN$3,800 for one drug, and perhaps more, because multiple medications are often required for the control of hypertension. This is an exceptional expense for many patients, but if we consider the large number of patients on antihypertensives, the cost to third parties is amplified remarkably. New drugs are generally more expensive than existing ones and therefore the lack of knowledge about the magnitude of the difference between less- and more-expensive drugs will continue to fuel the growth in drug expenditures as more new products come on the market.

Unfortunately, methodological weaknesses were common among the studies. Low response rates, convenience or unclear sampling, and unclear survey distribution are frequent weaknesses in cost awareness research, and these factors appeared to play a role in the study outcomes. While most studies had at least one methodological weakness, six (25%) of them had two or more weaknesses, and these studies seem to have a bias in favour of inflating doctors' cost awareness. Where only the higher-quality studies were used, physician awareness of costs became even poorer. Future research should focus on a large, defined sample of physicians (not a convenience sample), clarify the method of survey distribution, use methods to enhance response rates [27,54], and simultaneously survey doctors from different countries about a common group of drugs. There should be a clear description of true cost, use of comparable accuracy margins, and appropriate calculation of percent error (absolute numbers). Future authors should be aware of the bias associated with percent margins of error and the corresponding broader absolute margins with expensive drugs. They may want to consider doing additional analyses linking accuracy with decreasing margins of error for increasing true cost. It is important to correct these methodological deficiencies in order to be able to accurately determine if techniques to increase awareness of costs have had any influence on doctors' knowledge and behaviour.

While many people believe that doctors do not care about costs, secondary findings in the cost awareness studies show that doctors feel costs are important, and these findings are echoed in surveys of physicians' opinions of cost [55–57]. Swiss doctors feel costs, even to third-party payers, are important [55]. In the US, while 91% of doctors reported that costs are important when patients pay out-of-pocket, 80% felt that total (third-party) costs were also important [57]. A qualitative UK study found doctors give considerable thought to cost in their prescribing decisions in the context of ensuring quality of care [56]. Comments in some of the cost awareness studies indicate that doctors recognize the limits of their knowledge about costs, want more information, and feel that the provision of such information would reduce costs and either improve care or at least not negatively impact care. Despite this desire for more information and the potentially positive impact of providing it, doctors feel cost information is not accessible and some researchers even report significant challenges in obtaining cost information [35,44].

### Application of this Systematic Review

Studies in Israel [58,59] have demonstrated that in simulated cases, the addition of cost information modifies prescribing decisions in favour of reduced costs. A similar Canadian study [60] found that doctors did choose higher-cost drugs for patients with third-party coverage, but were sensitive to costs when provided and reduced expensive prescribing for covered drugs as well. These studies show the assumption that doctors are unconcerned about costs is mistaken. Rather, doctors feel that all costs (out-of-pocket and total) are important, they consider cost when prescribing and are sensitive to cost information. Therefore, more needs to be done to help physicians make costs part of their prescribing decisions.

Two studies [61,62] found that providing cost information alone as part of a program of computer prescribing did not reduce costs; however, in both cases the cost information was provided only once the drug choice was made, cost alternatives had to be sought, and in one [61], a relative score rather than the actual price was given. Furthermore, the before–after design led to bias because other coincidental initiatives caused inflation of drug costs during the intervention period [62].

Although data are limited, respondents in the cost awareness surveys indicated doctors' past cost education was limited, and in a recent US survey 89% of medical students indicated they wanted more education in health-care policy [63]. Clearly, more emphasis is required in the education of physicians about cost, and that education should likely start with improving the awareness of university faculty and medical school teachers. This is not to say doctors need to be educated about the cost of individual pharmaceuticals: As mentioned previously, that information varies dramatically and changes frequently, and physicians are already coping with information overload [64]. General cost education in medical school and residency should include an explanation of the large price differences between inexpensive and expensive drugs, the economies of time and scale, local coverage or copayment strategies, and perhaps that rising drug cost negatively impact funding to other areas.

In the clinical setting, providing cost information to doctors has had varying but generally positive results. Most studies show that providing cost information, particularly if combined with education and/or feedback, can modify prescribing and reduce costs [65–71]. Without directly providing cost information, other studies have shown that audit and feedback [72,73], educational interventions [74], and computerized prescribing with reminders [73,75] or evidence-based decision support [76] can all reduce costs.

Cost information could easily be incorporated into computerized prescribing software, giving doctors immediate information at the point of care. Physicians are rushed for time and typically spend less than two minutes to answer their clinical questions [77,78] so searching published documents or accessing other websites for cost information is not a realistic task. Even though some locations have cost information in prescribing software, it is not complete. For instance, in Australia where 98% of GPs use computer prescribing software [79], the pricing fields in the software are not uniformly filled in and limitations in the software may affect its utility [62].

While helpful, the measures proposed above are unlikely to have much impact in isolation, and most successful efforts in changing physician behaviour are multifactorial [80]. For instance, prescribing software does not present doctors with the relative costs of alternative treatments. If doctors are not aware that large differences can exist in the price of medications used for the same indication, they may continue to prescribe the more expensive varieties, especially if these drugs are being heavily promoted.

Therefore, real-time computerized prescribing software with cost information should also be linked with evidence-based decision support, which has been shown to reduce cost [76] as well as medication errors [81]. This information should be supplemented with real-time data on the relative costs of therapeutically equivalent medications. Additionally, physicians should also receive feedback and cost information and education, preferably personalized and rapid, similar to what has been delivered in the studies that have demonstrated reductions in prescribing costs [66,67,69,70]. Unfortunately, the most established audit–feedback system (PACT, or prescribing analysis and cost, in the UK) is mailed and arrives three to four months after the prescribing decisions have been made.

Future research should focus on programs that provide a combination of initial basic cost education, absolute and relative cost information integrated into point-of-care prescribing (preferably with evidence-based decision support), and ongoing audit–feedback and education. Due to expenses, audit–feedback and education would likely be most efficient if targeted on high-use drugs that have low-cost alternatives. The research should be randomized, prospective trials (not before/after) with control groups, and savings should be compared to program expenses.

### Limitations

A potential weakness of our study is the exclusion of non-English studies. From the abstracts of the non-English studies we identified, the estimation accuracy was 18% and 41%, indicating that inclusion would likely not have altered our findings significantly [82,83]. Only three of the studies included in our review were done after 1999, and consequently our results may not reflect current cost awareness of doctors. Of 24 studies, 21 came from the US, UK, and Canada. Knowledge of costs could be different in other countries, but studies from India, Italy, and Denmark had similar results and we that feel cost awareness would likely be similar in other countries.

## Supporting Information

Protocol S1 Systematic Review Protocol (35 KB DOC)

Table S1 The QUOROM Statement Checklist (56 KB DOC)

Table S2 Articles Excluded after Full Review and the Reason for Exclusion (91 KB DOC)

## Abbreviations

GP: general practitioner
