# Supplementary material for: Physician Awareness of Drug Cost: A Systematic Review
Source: PLoS Med. 2007 Sep 25;4(9):e283. doi: 10.1371/journal.pmed.0040283 (PMC1989748; doi:10.1371/journal.pmed.0040283)
Supplement: Protocol S1 — (35 KB DOC) [file pmed.0040283.sd001.doc]

**Search Strategy**

**Primary Database: PubMed / Medline**

**Search Terms:**

Physician OR physicians OR Doctor OR Medical Student OR House Staff OR intern OR Resident; AND

Medicine OR Medicines OR Medications OR drug OR therapeutic OR test OR investigation OR diagnostic test; AND

Cost OR Price; AND

knowledge OR awareness OR understanding

**Aditional Databases**

Embase

Econolit

Cochrane

**Additional Searching:**

Reference Review (any article pulled for possible inclusion)

Author Contact (authors with two or more publications in the area or who had published in the last 10 years)

Search done independently by both GMA and JL. Any article felt suitable by either reviewer included for full review.

**Inclusion/Exclusion**

**Inclusion Criteria**

1) Must be focused on Physicians Awareness of Medical Care Cost

- Physicians = Med students, residents, licensed practitioners

- Medical Care = Prescription Pharmaceuticals, Investigations, Hospital charges/consumables

2) Must have a description of how “True” or “Actual” costs determined

3) Cost awareness results described quantitatively

4) Clear description of how accuracy (knowledge/awareness) was quantified (e.g. “within +/-25% of actual). Accuracy may also be described as percent error, etc.

5) Must have > 10 participants/physicians

6) Each item (or aggregate of related items) estimated must have some data/results available (even in graphic form).

7) English Trials

**Exclusion Criteria**

1) Patient or non-physician cost awareness (exclude also those in which physician estimates can not be separated from non-physician)

2) Non prescription pharmaceuticals

3) Ranking or comparing the costs of medical care items

4) Studies in which participants estimated costs within ranges or cost increments (e.g. “Please estimate which $20 cost category/range is most appropriate for Drug A”)

Complete Independently (GMA & JL). Review results (GMA) and record any disagreement. GMA and JL discuss results to reach consensus.

**DATA ABSTRACTION FORM**

**Identification of Data Abstractor**

1. Initials of abstractor……………………………

**Identification of Study**

2. Record the first author’s last name, space, then initials

3. Record the journal name

4. Record the year of publication………………………………………….

1. Record the volume number……………………………………………..
2. Record the page numbers……………………………………………….
3. Publication status
   1. Full study
   2. Abstract
   3. Letter to the editor
4. Additional information obtained directly from author(s)
   1. Yes
   2. No

# Characteristics of study

1. Place where study took place
   1. Country
   2. State/province
   3. City
   4. Rural
2. Year when study done
3. Level of doctors studied and number of each (complete as many as appropriate):
   1. Fully licensed doctors
      1. Number enrolled (surveyed) in study
      2. Number completed study
      3. Response rate if actual numbers not given
   2. House staff (interns, residents, registrars) (no.)
      1. Number enrolled (surveyed) in study
      2. Number completed study
      3. Response rate if actual numbers not given
   3. Medical students (no.)
      1. Number enrolled (surveyed) in study
      2. Number completed study
      3. Response rate if actual numbers not given
4. For licensed doctors, no. of years in practice (percentages/group if available)
   1. Less than 5
   2. 5-10
   3. 10-20
   4. 20 +
5. Gender of doctors (% male)
6. Specialty of doctors (provide number/percentage per group, if available):
   1. General practice
   2. Other (specify)
7. Work site for doctors (provide number/percentage per group, if available):
   1. Hospital only
   2. Community only
   3. Hospital and community
8. Products involved (check as many as appropriate):
   1. Drugs/Therapeutics
   2. Investigations (e.g., x-rays)
   3. Medical supplies (e.g, iv equipment, bandages)
   4. Physician or hospital visit (or stay)
9. If drugs/therapeutics involved:
   1. Number of different drugs
   2. Number of different classes of drugs
10. If investigations involved,
    1. Number of Radiological
    2. Number of Laboratory
    3. Interventional and Other (specify),
11. If supplies involved, number of different types of supplies
12. If Physician or hospital visit (or stay) involved, number of different types (specify),
13. Method of ascertainment of true costs:
    1. From official price list (e.g., formulary)
    2. From actual acquisition cost by hospital or health authority
    3. Survey of pharmacies
    4. From manufacturers, wholesalers
14. Method of assignment of accuracy of results (complete all that apply):
    1. Within a certain percentage of true costs (specify percentage)
    2. Other (describe)
    3. Measure of accuracy (percent error, etc)
15. Unit of analysis
    1. Respondents
    2. Drugs/investigations/supplies
16. Accuracy (results within studies pooled with weighting by number of responses)
    1. If unit of analysis respondents, percent respondents
    2. If unit of analysis drugs/investigations/supplies/visit (percent for each separate item)
    3. If percent error (or other measures of accuracy) include,
    4. Other method of assessing accuracy,
17. Survey Methodology
    1. Sample selection
    2. Mode of Survey Distribution
18. Additional Information
    1. Percent of estimates above true costs:
    2. Percent of estimates below true costs:
    3. Miscellaneous information (qualitative)

Complete Independently. Review results (GMA) and record any disagreement. GMA and JL discuss results to reach consensus.

Statistical participation (NW) regarding potential combination of data and analysis.
