# Supplementary material for: Physician Awareness of Drug Cost: A Systematic Review
Source: PLoS Med. 2007 Sep 25;4(9):e283. doi: 10.1371/journal.pmed.0040283 (PMC1989748; doi:10.1371/journal.pmed.0040283)
Supplement: Table S1 — (56 KB DOC) [file pmed.0040283.st001.doc]

**Table S1. The QUOROM Statement Checklist**

| **Heading** | **Subheading** | **Descriptor** | **Reported? (Y/N)** | **Page Number** |
| --- | --- | --- | --- | --- |
| **Title** |  | Identify the report as a meta-analysis [or systematic review] of RCT's | Y | 1 |
| **Abstract** |  | Use a structured format | Y | 2 |
|  |  | **Describe** |  |  |
|  | Objectives | The clinical question explicitly | Y | 2 |
|  |  |  |  |  |
|  | Data Sources | The databases (ie. list) and other information sources | Y | 2 |
|  |  |  |  |  |
|  | Review methods | The selection criteria (ie. Population, intervention, outcome, and study design): methods of validity assessment, data abstraction, and study characteristics, and quantitative data synthesis in sufficient detail to permit replication | Y | 2 |
|  |  |  |  |  |
|  | Results | Characteristics of the RCTs included and excluded; qualitative and quantitative findings (ie. Point estimates and confidence intervals); and subgroup analyses | Y | 2-3 |
|  |  |  |  |  |
|  | Conclusion | The main results | Y | 3 |
|  |  |  |  |  |
|  |  | **Describe** |  |  |
| **Introduction** |  | The explicit clinical problem, biological rationale for the intervention, and rationale for review | Y | 4 |
|  |  |  |  |  |

**Table S1. The QUORUM Statement Checklist (continued)**

| **Methods** | Searching | The information sources, in detail (eg. Databases, registers, personal files, expert informants, agencies, hand-searching), and any restrictions (years considered, publication status, language of publication) | Y | 7-8 |
| --- | --- | --- | --- | --- |
|  |  |  |  |  |
|  | Selection | The inclusion and exclusion criteria (defining population, intervention, principal outcomes, and study design | Y | 8-9 |
|  |  |  |  |  |
|  | Validity assessment | The criteria and process used (eg. Masked conditions, quality assessment, and their findings) | Y | 9 |
|  |  |  |  |  |
|  | Data abstraction | The process or processes used (eg. Completed independently, in duplicate) | Y | 9 |
|  |  |  |  |  |
|  | Study characteristics | The type of study design, participants' characteristics, details of intervention, outcome definitions, &c, and how clinical heterogeneity was assessed | Y | 8-10 |
|  |  |  |  |  |
|  | Quantitative data synthesis | The principal measures of effect (eg. Relative risk), method of combining results (statistical testing and confidence intervals), handling of missing data; how statistical heterogeneity was assessed, a rationale for any a-priori sensitivity and subgroup analyses; and any assessment of publication bias | Y | 10-12 |

**Table S1. The QUORUM Statement Checklist (continued)**

| **Results** | Trial flow | Provide a meta-analysis profile summarizing trial flow (see figure) | Y | 12 (+ Figure 1) |
| --- | --- | --- | --- | --- |
|  |  |  |  |  |
|  | Study characteristics | Present descriptive data for each trial (eg. Age, sample size, intervention, dose, duration, follow-up period) | Y | 12-13 (+ Table 1) |
|  |  |  |  |  |
|  | Quantitative data synthesis | Report agreement on the selection and validity assessment; present simple summary results (for each treatment group in each trial, for each primary outcome); present data needed to calculate effect sizes and confidence intervals in intention-to-treat analyses (eg 2X2 tables of counts, means and SDs, proportions) | Y | 13-14 (+ Table 2&3 + Figure 2) |
|  |  |  |  |  |
| **Discussion** |  | Summarize key findings; discuss clinical inferences based on internal and external validity; interpret the results in light of the totality of available evidence; describe potential biases in the review process (eg. Publication bias); and suggest a future research agenda | Y | 16-17,  19-21 |
