# Supplementary material for: Physician Awareness of Drug Cost: A Systematic Review
Source: PLoS Med. 2007 Sep 25;4(9):e283. doi: 10.1371/journal.pmed.0040283 (PMC1989748; doi:10.1371/journal.pmed.0040283)
Supplement: Table S2 — (91 KB DOC) [file pmed.0040283.st002.doc]

**Table S2. Articles Excluded after Full Review and the Reason for Exclusion**

| **Citation** | **Reason for Exclusion** |
| --- | --- |
| Alastrue Loscos JI, Meneu de Guillerna R, Peiro Moreno S. (1998) Analysis of opinions, attitudes and knowledge of physicians of health centers of Valencia concerning efficacy and efficiency of drug prescriptions. Aten Primaria 21: 165-171. | Not English |
| Babington MA, Robinson LA, Monson RA (1983) Effect of written information on physicians’ knowledge of drug prices. Southern Medical Journal 76: 328-331,334. | Does not describe how actual cost determined. Analysis not done for individual drugs (all results pooled). |
| Barclay LP, Hatton RC, Doering PL, Shands JW Jr (1995) Physicians' perceptions and knowledge of drug costs: results of a survey. Formulary 30: 268-272, 277-279. | Drugs ranked in order of cost |
| Bustin J, Cassone J, Figueroa S. (2002) Mental Health Residents: Knowledge on prices and availability of drugs they prescribe. Vertex 13: 9-15. | Not English |
| Christo GG, Desai P, Krishna RC, Venkatesh A (1990) Knowledge of prescribers regarding cost of pediatric medications. Indian Pediatr 27: 83-85. | Does not explicitly say how true costs derived (implies that Indian Pharmaceutical Guide used) |
| Denig P, Haaijer-Ruskamp FM (1995) Do physicians take cost into account when making prescribing decisions? PharmacoEconomics 8: 282-290. | Review article |
| Denig P, Haaijer-Ruskamp FM, Wesseling H, Versluis A (1993) Drug expectations and drug choices of hospital physicians. J Int Med 234: 155-163. | Not Cost estimation. |
| Denig P, Haaijer-Ruskamp FM, Wesseling H, Versluis A (1993) Towards understanding treatment preferences of hospital physicians. Soc Sci Med 36: 915-924. | Not Cost estimation. |
| Diamond GA (1993) Doctor’s estimates of U.S. Health Care Spending. N Eng J Med 328: 1202. | Not a medical care item (the total health care spending) |
| Ernst ME, Kelly MW, Hoehns JD, Swegle JM, Buys LM, et al. (2000) Prescription medication costs: a study of physician familiarity. Arch Fam Med 9:1002-1007. | Price intervals |
| Fowkes F (1985) Doctor knowledge of the costs of medical care. Med Educ 19: 113-117. | Cost estimates were done by multiple choices from 4 possible prices |
| Forrest JB (1979) "Are doctors aware of hospital costs?" Va Med 106: 342-343. | Limited information on defining accuracy, true cost determination, etc |
| Frazier LM, Brown JT, Divine GW, Fleming GR, Philips NM, et al. (1991) Can physician education lower the cost of prescription drugs? A prospective, controlled trial. Ann Intern Med 115:116-121. | Comparison of costs (“which drug in this pair is more expensive”) not estimating actual costs |
| Freeman, RA (1976) Cost containment. J Med Educ 51: 157. | Unclear how actual costs determined. Otherwise data too limited (n = 4, types of tests unclear, no categorization of correct, etc) |
| Goldberg MA (1981) Cost of anticonvulsant therapy. Ann Neurol 9: 95. | No cost estimation |
| Gomez Perez B, Trilla Garcia A, Vernet Arias E, Corominas Garcia N, Codina Jane C, et al. (1996) Do physicians know the cost of the treatments and tests they prescribe? Rev Clin Esp 196: 523-528. | Used drug cost range |
| Griswold MW, Briceland LL, Lesar TS (1996) Costs of therapeutic alternatives: physicians' knowledge. Ann Pharmacother 30: 882-883. | Used drug cost ranges |
| Grubisich T (1979) Doctors fail quiz vs medical costs. Washington Post. Sept 21: B-4. | Unclear how actual costs determined. Otherwise data too limited (newspaper article). |
| Headly A (2002) Residents' knowledge of medication prices. Acad Med 77: 466. | Unclear how actual costs determined. Scale and Ranking of costs. |
| Hershey CO, Dawson NV, McLaren CE, Siciliano CJ, Cohen DI (1987) Resident knowledge of charges: are we asking the questions. Am J Med Sci 293: 182. | The estimates around drugs is pooled & the description around how cost are determined is weak (the cost to the institution). |
| Hoffman J, Barefield FA, Ramamurthy S (1995) A survey of physician knowledge of drug costs. J Pain Symptom Manage 10: 432-435. | Estimates were done by marking a line with cost increments, not by assigning actual numbers. There is very little data/information in the text. |
| Jager D, Baberg HT, Kugler J (2000) Who knows about costs in health care? Gesundh okon Qual manag 5: 80-88. | Does not describe how actual costs determined |
| Jayasuriya JP (1990) Cost awareness among junior anaesthetists in Sri Lanka. Ceylon Med J 35: 119-123. | Does not describe how actual cost determined (implies costs obtained from Department of Health) |
| Johnstone RE, Martinec CL (1994) Knowledge of medical charges. W V Med J 90: 226-229. | Does not describe how actual costs determined. Large portion is non-physician (85%) and can not separate out the medical. |
| Kaine RF, O’Connell, EJ (1978) Physicians’ appreciation of drug charges to the patient. Clin Pediatr 11: 665. | No quantifiable data (does not give information about results for different drugs) |
| Kelly SP (1978) Physicians’ knowledge of hospital costs. J Fam Pract 6: 171-172. | Used Cost Ranges (respondents asked to put items into predetermined cost categories) |
| Korn LM, Reichert S, Simon T, Halm EA (2003) Improving physicians' knowledge of the costs of common medications and willingness to consider costs when prescribing. J Gen Intern Med 18: 31-37. | Used Cost Ranges (respondents asked to put drugs into predetermined cost categories) |
| Kuiken T. Prather H (1998) A computer education program to improve physician awareness of rehabilitation hospital charges. Arch Phys Med Rehabil 79: 910-914. | Does not describe how actual costs determined. As well, Pretest, before feedback about costs, seems to have been presented in (and overlap with) Kuiken 1996. |
| Kuiken T, Prather H, Bloom S (1996) Physician awareness of rehabilitation costs. Am J Phys Med Rehabil 75: 416-421. | Does not describe how actual costs determined. Does not say which drugs used |
| Long MJ, Cummings KM, Frisof KB (1983) The role of perceived price in physician demand for diagnostic tests. Med Care 21: 243-250. | No indication how price determined. As well, limited presentation of data. |
| Lowy DR, Lowy L, Warner RS (1972) A survey of physicians' awareness of drug costs. J Med Educ 47: 349-351. | Limited data (2 drug costs estimated), limited presentation of data, inadequate description of how cost determined |
| Lyman JL, McCabe JB (1987) Emergency department care: cost awareness by health care providers. J Emerg Med 5: 567-571. | gives estimates of entire cost of patient care (Emergency) |
| Mader TJ, Playe SJ (1999) Academic emergency physicians' perception of patient charges resulting from routine ED care. Am J Emerg Med 17: 663-667. | Cost determination unclear. Individual items not identified distinctly but are separated in to groups (therapy, investigation, etc). |
| Marra C, Nimmo CR, Jewesson P (1995) A prospective survey of knowledge and perceptions of ondansetron: what do health care workers know about this drug? Can J Hosp Pharm. 48: 336-342. | Too few respondents (n=9), one drug only, multiple choice cost question. |
| McHugh T (1990) Cost awareness in an emergency medicine residency program. Ann Emerg Med 19: 494. | Unclear how costs determined and no data presentation. Does not give enough information about results for different drugs |
| Miller L, Blum A (1993) Physician awareness of prescription drug costs: a missing element of drug advertising and promotion. J Fam Pract 36: 33-36. | Respondents ask to put drugs into predetermined cost categories (ranges) |
| Mishra SK, Satpathy R (2001) Physicians' attitudes about prescribing and knowledge of the costs of common medications. Arch Intern Med 161: 1352-1353. | Very limited information, mixed MD/nurse, and n unknown. No indication of how true cost determined. |
| Mishra SK, Satpathy R (1999) Hospital staff do not know how much drugs cost. WJM 171: 225. | Very limited information, mixed MD/nurse. No indication of how true cost determined. |
| Nagurney JT, Braham RL, Reader GG (1979) Physician awareness of economic factors in clinical decision-making. Med Care 17: 727-736. | Does not describe how actual costs determined (implies that costs obtained from hospital pharmacy) |
| Petty C. Cost awareness is needed for cost-containment. AANA J. 1988; 56: 188-9. | Unclear how true costs determined. Does not give information about results for different drugs |
| Pugh JA, Frazier LM, DeLong E, Wallace AG, Ellenbogen P, et al. (1989) Effect of daily charge feedback on inpatient charges and physician knowledge and behavior. Arch Intern Med 149: 426-429. | Individual item estimates not available. |
| Reichert S, Simon T, Halm EA (2000) Physicians' attitudes about prescribing and knowledge of the costs of common medications. Arch Intern Med 160: 2799-2803. | Respondents ask to put drugs into predetermined cost categories |
| Reidenberg MM, Hodi FS (1991) A plea for prices in physician prescribing. JAMA 266: 3285. | No data |
| Robertson WO (1980) Costs of diagnostic tests: estimates by health professional. Med Care 18: 556-559. | Does not describe how actual costs determined |
| Roth R (1973) How well do you spend your patients’ dollars? Prism 1: 16. | Unclear cost determination. Unclear what constituted correct responses. |
| Ryan M, Yule B, Bond C, Taylor RJ (1996) Do physicians' perceptions of drug costs influence their prescribing? Pharmacoeconomics 9: 321-331. | Reanalysis of data presented in 1992 paper |
| Saunders AF, Divine GW, Weinberger M (1994) Physicians' awareness of mammography charges. Am J Prev Med 10: 357-360. | No estimation of therapeutics |
| Schnurrer JU, Stichtenoth DO, Troost R, Frolich JC (2001) Drug expenditure in hospitals: what do German ward physicians know? Br J Clin Pharmacol 51: 342-344. | Used cost ranges |
| Schroeder SL, Dobesh PP, Abu-Shanab JR, Lakamp JE (2002) Knowledge of hospital acquisition costs of antibiotics among physicians in a community hospital. Hospital Pharmacy 37: 833-839. | Inadequate presentation of data / description of findings. |
| Semel JD (1984) Physician cost awareness in prophylaxis and treatment of infection. IMJ Ill Med J 166: 157-160. | Not cost estimation. Number of respondents too low (n=7). |
| Shulkin DJ (1988) Cost estimates of diagnostic procedures. N Engl J Med 319:1291. | Does not describe how actual costs determined |
| Simpson PM (1978) The cost of anaesthetic drugs and equipment. Anaesthesia 33: 53-59. | Number of respondents too low (n=5) |
| Skipper JK, Smith G, Mulligan JL, Garg ML (1975) Medical students’ unfamiliarity with the costs of diagnostic tests. J Med Educ 50: 683-684. | Does not describe how actual costs determined. Limited presentation of data. |
| Skipper JK, Smith G, Mulligan JL, Garg ML (1976) Physicians’ knowledge of cost: the case of diagnostic tests. Inquiry 13: 194-198. | No estimations of therapeutics |
| Sumpton JE, Frewen TC, Rieder MJ (1992) The effect of physician education on knowledge of drug therapeutics and costs. Ann Pharmacother 26: 692-697. | No description of costs, multiple choices estimates (with results presented as scores) and limited data. |
| Thomas DR, Davis KM (1987) Physician awareness of cost under prospective reimbursement systems. Med Care 25: 181-184. | Does not describe how actual costs determined |
| Trinkaus J (1989) Estimating costs of diagnostic medical procedures: an informal look. Percept Mot Skills 69: 137-138. | Non-medical participants (no doctors) |
| Vosper HJ, Frewen TC (1993) Physician education and prescribing costs. Pharmacoeconomics 4: 77-84. | Basic Review article with limited data (none new). |
| Wolman RL, Abramson AW (1980) Students' and physicians' knowledge of hospital charges at Mount Sinai Hospital and School of Medicine. Mt Sinai J Med 47: 568-574. | Does not provide information about individual items |
| Yule B, Ryan M, Bond C, Taylor RJ (1991) Awareness of drug costs among general practitioners trainees. Med Educ 25: 536-538. | Does not describe how actual costs determined |
| Zelnio RN, Gagnon JP (1979) The effects of price information on physician prescribing patterns – a literature review. Drug Intelligence and Clinical Pharmacy 13: 156-159. | Basic review article. |
